# Merkel Cell Polyomavirus Small T Antigen Promotes Pro-Glycolytic Metabolic Perturbations Required for Transformation

**DOI:** 10.1371/journal.ppat.1006020

**Published:** 2016-11-23

**Authors:** Christian Berrios, Megha Padi, Mark A. Keibler, Donglim Esther Park, Vadim Molla, Jingwei Cheng, Soo Mi Lee, Gregory Stephanopoulos, John Quackenbush, James A. DeCaprio

**Affiliations:** 1 Department of Medical Oncology, Dana-Farber Cancer Institute, Boston, Massachusetts, United States of America; 2 Program in Virology, Graduate School of Arts and Sciences, Harvard University, Cambridge, Massachusetts, United States of America; 3 Department of Biostatistics and Computational Biology, Dana-Farber Cancer Institute, Boston, Massachusetts, United States of America; 4 Department of Biostatistics, Harvard School of Public Health, Boston, Massachusetts, United States of America; 5 Department of Chemical Engineering, Massachusetts Institute of Technology, Cambridge, Massachusetts, United States of America; 6 Department of Medicine, Brigham and Women’s Hospital, Harvard Medical School, Boston, Massachusetts, United States of America; 7 Department of Medicine, Harvard Medical School, Boston, Massachusetts, United States of America; University of Wisconsin-Madison, UNITED STATES

## Abstract

Merkel cell polyomavirus (MCPyV) is an etiological agent of Merkel cell carcinoma (MCC), a highly aggressive skin cancer. The MCPyV small tumor antigen (ST) is required for maintenance of MCC and can transform normal cells. To gain insight into cellular perturbations induced by MCPyV ST, we performed transcriptome analysis of normal human fibroblasts with inducible expression of ST. MCPyV ST dynamically alters the cellular transcriptome with increased levels of glycolytic genes, including the monocarboxylate lactate transporter SLC16A1 (MCT1). Extracellular flux analysis revealed increased lactate export reflecting elevated aerobic glycolysis in ST expressing cells. Inhibition of MCT1 activity suppressed the growth of MCC cell lines and impaired MCPyV-dependent transformation of IMR90 cells. Both NF-κB and MYC have been shown to regulate MCT1 expression. While MYC was required for MCT1 induction, MCPyV-induced MCT1 levels decreased following knockdown of the NF-κB subunit RelA, supporting a synergistic activity between MCPyV and MYC in regulating MCT1 levels. Several MCC lines had high levels of MYCL and MYCN but not MYC. Increased levels of MYCL was more effective than MYC or MYCN in increasing extracellular acidification in MCC cells. Our results demonstrate the effects of MCPyV ST on the cellular transcriptome and reveal that transformation is dependent, at least in part, on elevated aerobic glycolysis.

## Author Summary

In 2008, Merkel cell polyomavirus (MCPyV) was identified as clonally integrated in a majority of Merkel cell carcinomas (MCC), a rare but highly aggressive neuroendocrine carcinoma of the skin. Since then, studies have highlighted the roles of the MCPyV T antigens in promoting and sustaining MCC oncogenesis. In particular, MCPyV small T antigen (ST) has oncogenic activity *in vivo* and *in vitro*. We performed transcriptome analysis of normal human fibroblasts with inducible expression of MCPyV ST and observed significant alterations in levels of metabolic pathway genes, particularly those involved in glycolysis. MCT1, a major monocarboxylate transporter, was rapidly induced following ST expression and inhibition of MCT1 activity reduced the ST growth promoting and transforming activities. The metabolic perturbations induced by this oncogenic human polyomavirus reflect a potent transforming mechanism of MCPyV ST.

## Introduction

Human polyomaviruses are a diverse family of small DNA tumor viruses that typically cause asymptomatic, lifelong infections in healthy individuals [1, 2]. However, immune deficiencies enable more severe polyomavirus induced diseases including Merkel cell carcinoma (MCC). MCC is a rare and aggressive skin cancer that primarily affects the elderly and immunocompromised [3, 4]. Transcriptome sequencing of MCC led to the discovery of Merkel cell polyomavirus (MCPyV) and demonstration that viral DNA was clonally integrated in approximately 80% of MCC tumors [5]. The integrated MCPyV early-region (ER) expresses wild-type small T antigen (ST) and a truncated form of large T antigen (LT). The truncated LT retains the LXCXE motif that binds the retinoblastoma protein (RB1) but is unable to support viral replication due to loss of the DNA binding and helicase domains [6, 7]. In some MCC tumors, ST can be detected in the absence of LT, suggesting that ST is required for tumorigenesis [7].

The precise mechanisms for how MCPyV ST promotes cellular transformation are still unresolved. The contribution of the related polyomavirus simian virus 40 (SV40) ST to transformation has been shown to be dependent on its ability to bind and inhibit protein phosphatase 2A (PP2A) activity that, in turn, perturbs a wide range of signaling pathways [8, 9]. However, PP2A binding by MCPyV ST may not be necessary for transformation [7]. Compared to ST from other polyomaviruses, MCPyV ST has unique properties including inhibition of the E3 ubiquitin ligase FBXW7 and an ability to increase cap-dependent translation through hyperphosphorylation of 4E-BP1 [10–12], activities which likely contribute to the initiation and maintenance of an oncogenic state.

Under normal physiological conditions, cells can convert one glucose molecule into two pyruvate molecules followed by pyruvate oxidation in mitochondria resulting in the synthesis of 38 ATP molecules per molecule of glucose [13]. In hypoxic conditions, oxidative phosphorylation is inhibited and anaerobic glycolysis is activated, leading to the production of only 2 ATP molecules and secretion of lactate into the extracellular space [14]. Cancer cells may convert pyruvate to lactate under normoxic conditions resulting in aerobic glycolysis, known as the Warburg effect. Increased aerobic glycolysis has been recognized as a hallmark of cancer due to the requirement for large quantities of biosynthetic intermediates to be generated for sustained tumorigenesis [15, 16]. Excess lactate production increases the acidity of the tumor cell microenvironment that favors two additional hallmarks, tumor cell invasion and metastasis [17].

Viruses typically do not express their own metabolic enzymes and instead rely on manipulation of host signaling and metabolic pathways to establish productive infections [18]. For example, RNA viruses such as hepatitis C and dengue virus promote efficient replication by altering host lipid metabolism [19]. Adenovirus can perturb the function of the transcription factor MYC to promote glucose and glutamine metabolism [20], and Kaposi’s Sarcoma-associated Herpesvirus (KSHV) has also been shown to induce MYC expression leading to increased glutaminolysis [21].

To gain insight into the impact of MCPyV ST on gene expression, we generated normal human cells with inducible expression of MCPyV ST or GFP and performed transcriptome profiling. The resulting analysis revealed that ST significantly altered metabolism-related pathways with upregulation of glycolysis and metabolite transporter genes. Correspondingly, we found that increased ST levels led to increased aerobic glycolysis. Furthermore, inhibition of the major monocarboxylate transporter MCT1 suppressed ST induced cellular proliferation, transformation and MCC viability.

## Results

### MCPyV ST dynamically alters the transcriptome of normal human cells

**Fig 1:**
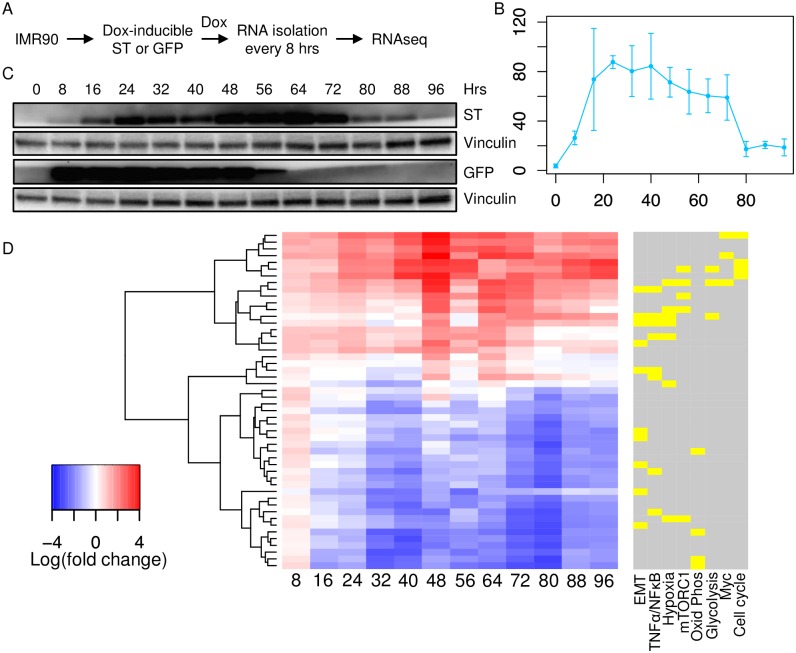
Temporal transcriptome of IMR90 fibroblasts inducibly expressing MCPyV ST. **A)** IMR90 fibroblasts containing dox-inducible MCPyV ST or GFP vectors were treated with dox and harvested every 8 hours for RNA extraction. Each time point represents three biological replicas. **B)** Mean ST transcript levels and **C)** immunoblotting for ST, GFP and vinculin from cells collected every 8 hours for 96 hours following dox treatment. **D)** Hierarchical clustering and fold change between MCPyV ST and GFP following dox induction for 96 hours. Each bar represents an average of three experiments for each time point. The enrichment of “Cancer Hallmark” gene sets are represented relative to the ST-differentially expressed clusters, including epithelial to mesenchymal transition (EMT), tumor necrosis factor-α (TNFA signaling via NF-κB), hypoxia, mTORC1, oxidative phosphorylation, glycolysis, MYC, and several cell cycle clusters including E2F targets, G2M checkpoint and mitotic spindle. The color bar indicates statistical significance, yellow p < 0.05 and gray p > 0.05.

Given the unique properties of MCPyV ST compared to ST from other human polyomaviruses [1, 11], we sought to obtain a global view of MCPyV ST induced transcriptional perturbations in normal human cells. IMR90 human diploid lung fibroblasts were selected because of their wide use in transcriptome and genomic analyses, including viral oncoprotein perturbations [22]. Cells were transduced with lentiviral vectors capable of doxycycline (dox) inducible MCPyV ST or GFP expression. Cells were treated with dox for 96 hours and harvested every 8 hours followed by processing for RNAseq in triplicate (Fig 1A). ST transcript levels were rapidly induced with peak levels between 24 to 40 hours after dox addition, followed by a gradual decrease in levels (Fig 1B). ST protein levels followed the transcript profile, peaking between 48 to 64 hours before declining (Fig 1C). Similarly, GFP protein levels were detectable within 8 hours and decreased by 64 hours after dox addition.

Genes that were most differentially expressed between ST and GFP were ranked by LIMMA (see Methods) with 2854 genes passing p-value and fold-change cutoffs [23]. Model-based clustering grouped the differentially expressed genes into 50 clusters (Fig 1D and S1 Fig and S1 Table). Clusters were evaluated for significant enrichment in biological processes, including Gene Ontology (GO) terms (S2 Table) and the Cancer Hallmark gene sets in the Molecular Signatures Database (MSigDB) (S3 Table) [24]. ST expressing cells were significantly enriched for gene clusters with the GO terms for energy coupled proton transport, isoprenoid and L-serine biosynthetic processes, and glutamine, lysine and arginine transport. ST expressing cells were also significantly enriched for the Cancer Hallmarks including epithelial to mesenchymal transition (EMT), TNFA signaling via NF-κB, hypoxia, mTORC1, oxidative phosphorylation, glycolysis, MYC, and cell cycle including E2F targets, G2/M checkpoint and mitotic spindle.

### MCPyV ST upregulates glycolytic and metabolite transport genes including the major monocarboxylate transporter SLC16A1

**Fig 2:**
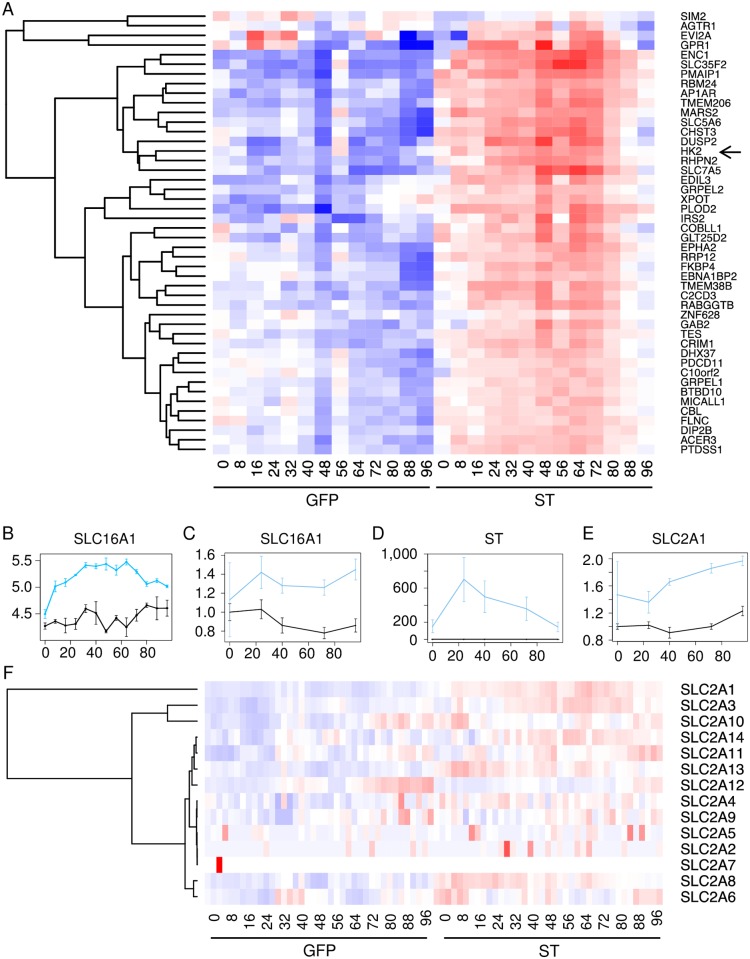
MCPyV ST induces metabolism-related genes and SLC16A1. **A)** Cluster 6 highlights differentially expressed metabolic enzymes. Arrow indicates HK2 gene expression profile. **B)** Mean transcript levels (log_2_FPKM) of the SLC16A1 (MCT1) monocarboxylate transporter after induction of ST (blue) or GFP (black) expression for 96 hours. RT-qPCR evaluation of SLC16A1 (**C**), ST (**D**) and SLC2A1 (GLUT1) (**E**) levels in GFP (black line) and ST (blue) induced cell lines at 0, 40, 72 and 96 hours. **F**) Differential expression of GLUT hexose transporters (SLC2A1-14).

Elevated aerobic glycolysis is a hallmark present in many cancers and represents a potential vulnerability for targeting cancer cell proliferation [15, 16, 25]. Expression profiling of MCPyV ST cells revealed a high prevalence of significantly altered metabolism-related genes, specifically those involved in glycolysis such as Hexokinase 2 (HK2) in cluster 6 (Fig 2A) and metabolite transport. Transport of metabolites is mediated in large part by members of the SLC gene family [26]. A large proportion of all SLC transporter genes were significantly upregulated following ST expression (S2 Fig). A consequence of increased aerobic glycolysis is elevated acidification of the surrounding microenvironment. Notably, expression of SLC16A1 (MCT1), the major monocarboxylate transporter for lactate and pyruvate [27, 28], was significantly increased after ST induction (Fig 2B and 2C). Levels of MCT1 were increased in IMR90 cells expressing ST compared to GFP following dox addition.

Activation of glycolysis is normally accompanied by an increase in the rate of glucose import. Consistent with this, we found that MCPyV ST increases the expression of two glucose transporters, GLUT1 (SLC2A1) and GLUT3 (SLC2A3). We validated the increase in the expression of GLUT1 in ST expressing using RT-qPCR (Fig 2E). Among the larger family of hexose transporters (SLC2A1-14) we also observed upregulation of SLC2A8, 13, and 14 (Fig 2F). Additionally, we found that ST cells have higher expression of the carbohydrate response element binding proteins (ChREBPs) MLX and MLXIP, which can bind and activate the promoters of genes encoding glycolytic enzymes, thus increasing the rate of glycolysis (S3A Fig).

### MCPyV ST expressing cells exhibit aerobic glycolysis and MCT1 sensitivity

**Fig 3:**
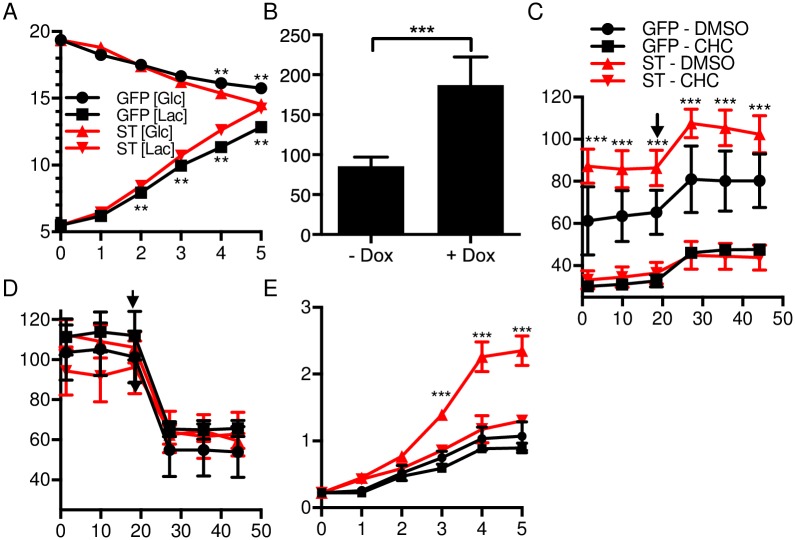
MCPyV ST increases aerobic glycolysis and MCT1 sensitivity. **A)** Media glucose (Glc) and lactate (Lac) levels (mM) from cultures of IMR90 cells expressing ST or GFP were measured at the indicated day following dox addition. **P < 0.005 calculated using unpaired student’s T test between the marked GFP and ST points. **B)** ECAR (mpH/min) of IMR90 cells inducibly expressing ST with and without dox addition for 48 hours. ***P < 0.0005 calculated using unpaired student’s T test. **C)** ECAR of IMR90 cells expressing ST or GFP with CHC (5 mM) or DMSO (minutes) following 48 hours of dox treatment. Cells were treated with oligomycin (1 μM) at the indicated time point. ***P < 0.0005 calculated using unpaired student’s T test between GFP-DMSO and ST-DMSO samples. **D)** OCR (pmoles/min) of cells (minutes) as in C. **E)** Growth of IMR90 cells expressing ST or GFP treated with dox and CHC or DMSO was assessed by crystal violet every day for 5 days. ***P < 0.0005 calculated using unpaired, two-tailed student’s T test between ST-DMSO and ST-CHC treatments. Key same as in C.

Glycolysis is a multistep biochemical process. HK2 serves as an upstream regulator that irreversibly commits glucose to enter the pathway. A byproduct of aerobic glycolysis is lactate. Production of lactate from pyruvate is mediated by lactate dehydrogenase (LDH), comprised of homo- or heterotetramers of two subunits, LDHA and LDHB. A major function of MCTs, including MCT1, is to prevent the toxic buildup of lactate in the intracellular milieu by co-exporting lactate together with protons out of the cell [27]. We observed that levels of glucose were depleted from the media and lactate levels increased at a significantly higher rate following ST expression compared to GFP cells (Fig 3A).

Given the significant increase in lactate levels in the media as early as 2 days after ST induction, we measured the extracellular acidification rate (ECAR) of IMR90 cells inducibly expressing ST before and after dox addition for 48 hours. We found a significant increase in the ECAR of these cells following ST induction, consistent with an increase in aerobic glycolysis (Fig 3B and 3C). Inhibition of ATP synthase by oligomycin treatment led to an increase in the glycolytic rate of cells in response to the lack of ATP production from oxidative phosphorylation (Fig 3C) [29]. As expected, we found the ECAR to be significantly decreased in both ST and GFP expressing cells treated with the MCT inhibitor α-cyano-4-hydroxycinnamate (CHC) (Fig 3C).

There was no significant difference in the oxygen consumption rate (OCR) of ST cells compared to GFP cells (Fig 3D). This suggests that the level of oxidative phosphorylation is maintained in ST cells despite the increased rate of glucose being converted to lactate. ST cells may use alternative carbon sources, like glutamine, to fuel the TCA cycle. Consistent with this hypothesis, we found that ST cells upregulate the expression of the glutamine transporter SLC1A5, as well as the enzymes glutaminase (GLS) and glutamate dehydrogenase (GLUD1), which are necessary to convert glutamine to the TCA cycle intermediate α-ketoglutarate through the metabolic pathway called glutaminolysis (S3B Fig).

To determine the importance of MCT1 activity on the fitness of ST expressing cells, we measured the growth of IMR90 cells expressing either ST or GFP following CHC or DMSO treatment (Fig 3E). CHC significantly suppressed the growth rate of ST expressing cells, while GFP cells were largely unaffected.

### MCC cell lines exhibit variable ECAR that correspond to MCT1 inhibitor sensitivity

**Fig 4:**
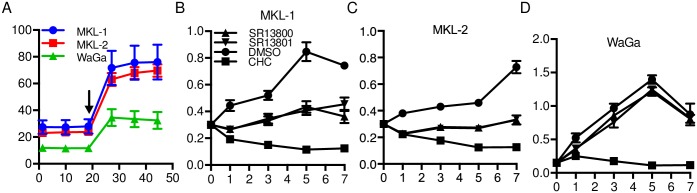
MCC cell lines exhibit variable ECAR and sensitivity to MCT1 inhibition. **A)** ECAR (mpH/min) of MKL-1, MKL-2 and WaGa lines (minutes). Cells were treated with oligomycin (1 μM) at the indicated time point (arrow). **B-D)** XTT proliferation assay of MKL-1, MKL-2 and WaGa cells treated with either DMSO, CHC (5 mM), SR13800 (100 nM), or SR13801 (100 nM) (days).

We assessed the glycolytic pathway in three MCPyV-positive MCC cell lines. ECAR measurements revealed that MKL-1 and MKL-2 cells had similar rates of glycolysis, while WaGa cells had a lower glycolytic rate (Fig 4A). Conversely, following oligomycin treatment, MKL-1 and MKL-2 cells increased their ECAR levels higher than WaGa cells consistent with their higher level of glycolysis. Inhibition of MCT1 in a highly glycolytic cell can lead to intracellular acidification through the accumulation of monocarboxylates and protons. MCT1 inhibition has previously been shown to be toxic to certain tumors with high MCT1 expression [27, 30]. A number of MCT1 inhibitors are current in clinical trials for treating advanced solid tumors, with promising results in cancers with elevated MCT1 expression [31, 32]. SR13800 and SR13801 are pyrole pyrimidine-based molecules with high specificity for MCT1 [33]. We tested the effects of the MCT1 inhibitors CHC, SR13800 and SR13801 on the viability of MKL-1, MKL-2 and WaGa MCC cell lines over 7 days (Fig 4B, 4C and 4D, respectively). All three MCC cell lines showed high sensitivity to CHC treatment, while only MKL-1 and MKL-2 cells were affected by SR13800 and SR13801. MKL-1 and MKL-2 cells were more dependent than WaGa cells on MCT1 activity and glycolysis for continued proliferation reflecting their higher ECAR levels.

### MYC isoforms differentially regulate aerobic glycolysis in MCC cell lines

The expression of HK2, LDH, MCT1 and other glycolytic genes are regulated, at least in part, by MYC [25, 34]. Given the pronounced effects of ST on glycolysis, we sought to determine how sensitive MCPyV-positive MCC cell lines were to perturbations in this pathway. Since the MCC cell lines MKL1, MKL-2, WaGa and BroLi were previously uncharacterized with regards to MYC, we assessed the levels of the different MYC isoforms in these cells. By immunoblotting, these MCC cell lines had no detectable MYC, but did have detectable levels of MYCN and MYCL (S4A Fig).

**Fig 5:**
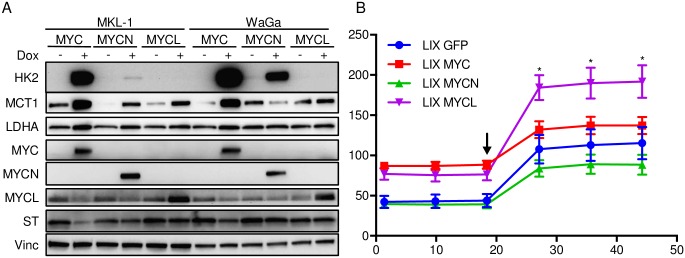
MYC isoforms differentially regulate glycolysis gene expression and ECAR of MCC cells. **A)** MKL-1 and WaGa cells containing inducible vectors for MYC, MYCN or MYCL were treated with (+) or without (-) dox for 72 hours and lysates were immunoblotted with the indicated antibodies. **B)** ECAR (mpH/min) of MKL-1 cells inducibly expressing GFP, MYC, MYCN or MYCL after 72 hours of dox addition (minutes). Cells were treated with oligomycin (1 μM) at the indicated time point. *P < 0.05 calculated using unpaired student’s T test between MYC and MYCL samples.

We next determined the ability of each MYC family member to regulate glycolytic gene expression and aerobic glycolysis in MCC cell lines. MKL-1 and WaGa cells were transduced with dox inducible vectors expressing MYC, MYCN or MYCL. Following selection, cells were treated with dox for 72 hours, and then lysates were harvested for immunoblotting (Fig 5A). We observed that MYC and MYCN but not MYCL led to increased levels of HK2 and LDHA in MKL-1 and WaGa cells. MYC and MYCL led to increased levels of MCT1 while MYCN led to decreased MCT1 levels in WaGa cells.

Given how the various MYC isoforms differentially affected HK2, LDHA and MCT1 levels, we compared the effects of MYC expression in MKL-1 cells on aerobic glycolysis by measuring the ECAR following 72 hours of dox treatment (Fig 5B). GFP and MYCN expressing cells had similar basal ECAR, while MYC and MYCL expressing cells had higher ECAR levels. Notably, the MYCL expressing MKL-1 cells had significantly higher ECAR following oligomycin treatment, indicating that MYCL was more effective in facilitating the glycolytic capacity of these cells compared to the other MYC isoforms.

### MYC and NF-κB have differential effects on MCPyV-driven glycolytic gene induction

**Fig 6:**
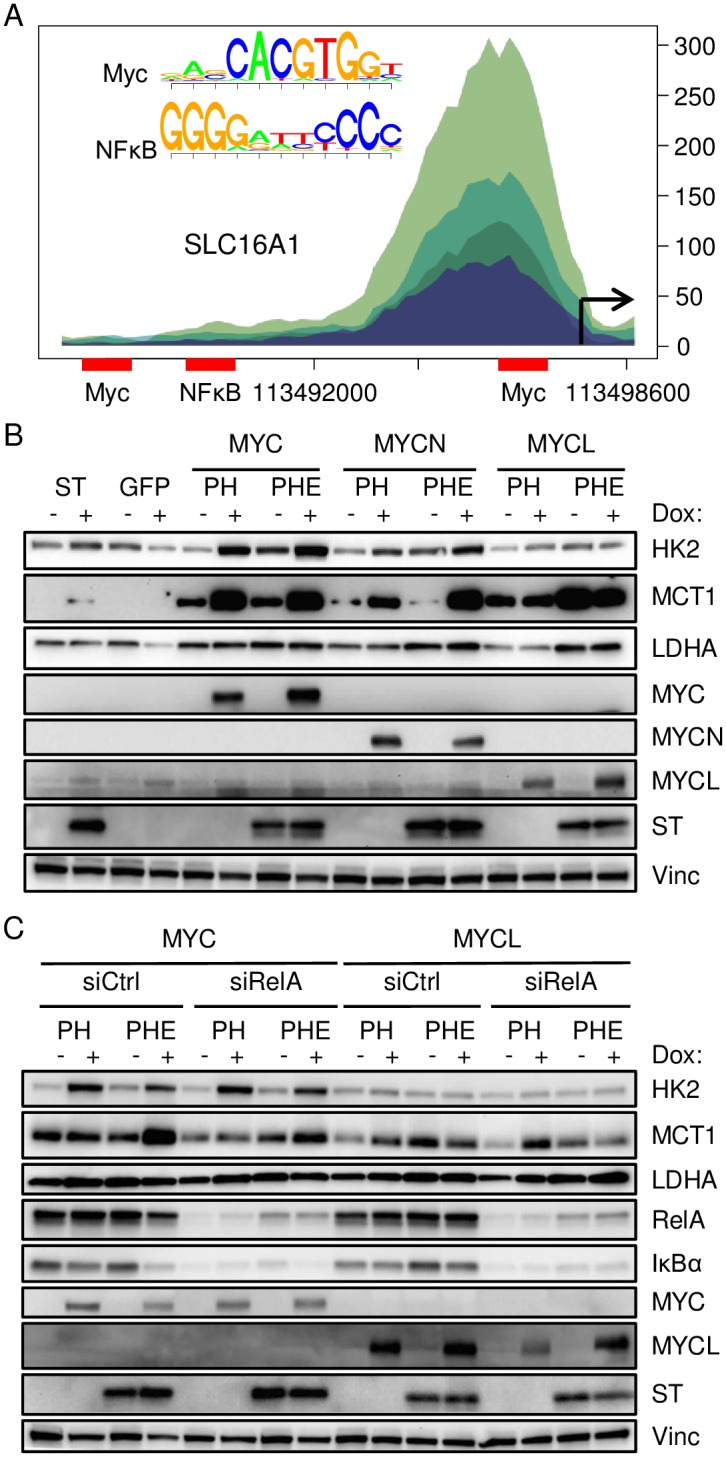
Differential effects of MYC and NF-κB on glycolytic gene and MCT1 levels. **A)** Promoter region of MCT1 includes MYC and NF-κB binding sites. Shading reflects four independent IMR90 DNase I hypersensitivity datasets. **B)** IMR90 cells stably expressing ST, GFP or p53DD + hTERT (PH) and MCPyV tumor-derived early-region (PHE) with inducible expression of MYC, MYCN or MYCL were treated with dox (+) for 48 hours. Lysates were immunoblotted with the indicated antibodies. **C)** IMR90 PH and PHE cells inducibly expressing MYC or MYCL were transfected with RelA-specific pooled siRNA (siRelA) or non-targeting siRNA (siCtrl). After 24 hours, cells were refed with dox containing media and lysed after an additional 48 hours.

We sought to identify specific signaling pathways that ST utilized to increase glycolytic and MCT1 gene expression and if these pathways contributed to cellular transformation. Given the significant enrichment of genes from both the MYC and NF-κB Cancer Hallmark gene sets across several ST induced gene clusters (Fig 1D), we examined the MCT1 promoter region [-1000, +100] for MYC and NF-κB binding sites (Fig 6A). Chromatin accessibility patterns in the parental IMR90 cells were assessed in the ENCODE DNase I hypersensitivity experiments (GEO: GSM468801, GSM530665, GSM530666, GSM468792). A MYC binding site was located within a relatively open region in the MCT1 promoter, while a NF-κB binding site was located in an area of reduced hypersensitivity.

To investigate the regulation of glycolysis gene expression in the context of a MCPyV-transformed cell line, IMR90 cells were serially transduced with retroviral constructs expressing a dominant-negative form of p53 (p53DD), telomerase reverse transcriptase (hTERT), and a tumor-derived form of MCPyV early-region (ER) that expresses ST plus truncated LT to generate p53DD-hTERT-ER (PHE) cells. Unlike SV40 large T antigen (LT), MCPyV LT cannot bind and inhibit p53; therefore p53DD is required to bypass senescence and apoptotic checkpoints in these IMR90 cells [35, 36].

To determine if MYC family proteins could cooperate with MCPyV IMR90 cells, we treated cells stably expressing ST, GFP, or p53DD + hTERT (PH) and MCPyV tumor-derived early-region (PHE) with inducible expression of MYC, MYCN or MYCL with dox for 48 hours and immunoblotted for HK2, MCT1 and LDHA (Fig 6B). ST alone could induce MCT1 levels and both PH and PHE cells had higher levels of MCT1 expression than ST alone. Consistent with the effects seen in MCC cell lines (Fig 5A), MYC and MYCN induction but not MYCL led to increased levels of HK2 and MCT1 in PH and PHE cells. The presence of MCPyV ER did not appear to affect induction of HK2 or MCT1 by MYC or MYCN. In contrast, PHE cells with MYCL had very high levels of MCT1 protein in the uninduced state, likely due to leakiness of the vector (S4B and S4C Fig). These results indicate that MYCL but not MYC or MYCN was able to cooperate with MCPyV ER to induce MCT1.

In addition to MYC, NF-κB is a prominent inducer of metabolic and growth-promoting genes and has been shown to independently regulate MCT1 [33, 37, 38]. RelA, also known as p65, is a key subunit of canonical NF-κB signaling and forms a homo- or heterodimer with other NF-κB subunits to activate target genes including IκBα [39]. To assess the role of NF-κB in the regulation of MCT1 in the context of MCPyV, IMR90 PH and PHE cells with inducible MYC or MYCL were transfected with siRNA targeting RelA (siRelA) or a non-targeting control (siCtrl). Cells were re-fed with or without dox-containing media 24 hours after transfection, then lysed 48 hours later for immunoblotting (Fig 6C). Following siRelA but not siCtrl treatment, reduced levels of RelA and the downstream target IκBα were observed in both MYC and MYCL expressing PH and PHE lines. Knockdown of RelA did not affect the ability of MYC to induce HK2 in the PH and PHE cells. In contrast, MCT1 levels were reduced following knockdown of RelA across all conditions, indicating that canonical NF-κB signaling likely has a complementary role in regulating MCT1 levels.

### Targeting MCT1 activity inhibits MCPyV-induced transformation

**Fig 7:**
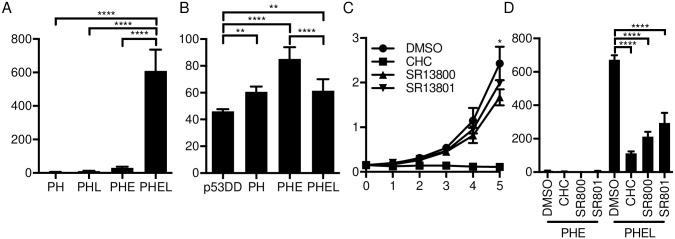
MCPyV-transformed cells exhibit elevated ECAR and sensitivity to MCT1 inhibitors. **A)** Anchorage-independent growth of IMR90 PH, PHL, PHE and PHEL cells. ****P < 0.0001 calculated using ordinary one-way ANOVA with multiple comparisons. **B)** Basal ECAR (mpH/min) measurement of p53DD, PH, PHE and PHE + MYCL (PHEL) cells. **P < 0.005 and ****P < 0.0001 calculated using ordinary one-way ANOVA with multiple comparisons. **C)** Proliferation of PHEL cells treated with DMSO, CHC (5 mM), SR13800 (100 nM), or SR13801 (100 nM) was assessed by crystal violet staining. *P < 0.05 calculated using student’s T test between DMSO-SR13800 and DMSO-SR13801 samples. **D)** Anchorage-independent growth of IMR90 PHE and PHEL cells treated with DMSO, CHC, SR13800 (SR800) or SR13801 (SR801). ****P < 0.0001 calculated using ordinary one-way ANOVA with multiple comparisons.

We assessed whether MCPyV-mediated transformation could be attenuated by MCT1 inhibition. We found that overexpression of MYCL in PHE (PHEL) IMR90 cells led to robust IMR90 anchorage-independent growth in soft agar, while PH, PHL and PHE cells lacked significant colony formation (Fig 7A). We chose MYCL in this context as it was previously shown that MYCL is amplified in MCC tumors, and may therefore have oncogenic potential in the presence of MCPyV [40]. We measured basal ECAR of IMR90 cells stably expressing p53DD, PH, PHE and PHEL (Fig 7B) and found that PH, PHE and PHEL cells had significantly higher ECAR than p53DD cells, with PHE cells maintaining the highest rate while PHEL cells had a significantly lower ECAR than PHE cells.

To determine if MCT1 activity was necessary to support cellular transformation of IMR90 cells by MCPyV, we assessed anchorage-independent growth by culturing PHE and PHEL cells in soft agar in the presence of the MCT1 inhibitors or DMSO. Proliferation of PHEL cells was highly attenuated by CHC treatment, while SR13800 and SR13801 inhibitors had modest but significant effects on growth compared to DMSO treatment (Fig 7C). PHEL cell growth in soft agar was significantly inhibited by CHC, SR13800 and SR13801 compared to DMSO treated cells (Fig 7D). These results indicate that MCT1 activity was required for transformation of IMR90 cells by MCPyV and MYCL.

## Discussion

Metabolic perturbations represent a key hallmark in many cancers, as the energetic and biosynthetic demands of tumor cells increase to sustain proliferation. MCPyV ST is a potent oncoprotein with transforming potential *in vitro* and *in vivo* and contributes to MCC. By performing temporal transcriptional profiling and metabolic analysis of ST expressing cells, we determined that ST significantly increases aerobic glycolysis and that inhibition of this pathway can suppress MCPyV-induced transformation as well as MCC growth. Cancers with viral etiology are particularly likely to undergo metabolic alterations due to the fundamental need for viruses to create a pro-replicative environment. Many viruses, including adenovirus, hepatitis C virus and HIV, induce aerobic glycolysis in infected cells to support viral replication [18]. Our results indicate that MCPyV ST can specifically alter the metabolic state of a cell.

We designed a time-series RNA-sequencing experiment to characterize the dynamics of gene expression in cells after expression of MCPyV ST. Comparing with statistically distinct behavior in the ST-expressing cells relative to GFP-expressing cells, we found that most of the differential expression trends appeared already at 16 hours post-induction, with down-regulated genes first reaching a minimum at around 32 hours and up-regulated genes building more gradually to peak at the 48 hour mark. Most genes exhibited only down- or up-regulation throughout the time course of 96 hours. We grouped differentially expressed genes into clusters to build a global picture of how ST remodels the transcriptional landscape. Among the 50 resulting clusters and their GO term and pathway enrichment, we observed a strong signature of metabolism-related changes (Fig 1D and S1 Fig). Many of the up-regulated clusters were enriched for the glycolysis pathway, rRNA processing, amino acid transport and response to glucose starvation. Among down-regulated clusters, there was enrichment in fatty acid oxidation, purine and pyrimidine metabolic processes, lipid metabolism, and mitochondrial respiration and ATP synthesis genes. The transcriptional signature of ST-expressing cells exhibited many of the characteristics associated with activation of aerobic glycolysis. In particular, we found that ST upregulated glucose import, lactate export and ChREBPs, transcription factors that specifically activate glycolytic enzymes. In addition, we found evidence that ST cells maintain normal levels of oxidative phosphorylation through anaplerosis, through increased levels of glutamine transporter and GLS and GLUD1, critical for glutaminolysis.

MCPyV ST also induced changes in many genes that were not annotated to be involved in metabolic processes. There were 14 out of the 50 clusters that showed enrichment for GO terms not involved in metabolism (S2 Table). The up-regulated genes were enriched for the mitotic cell cycle (clusters 46, 12, 31, 39), SMAD and BMP pathways (clusters 23 and 15), vascular permeability (3), keratinocyte migration (9) and pinocytosis (14). The down-regulated genes were involved in synapse assembly (19), SMAD protein import into the nucleus (43), extracellular matrix organization (38), cell adhesion (24), and negative regulation of viral genome replication (40). Therefore, ST appears to have a major impact on genes in metabolic and cell cycle pathways as well as several additional transcriptional programs, including the SMAD/BMP differentiation pathway and cell adhesion.

Given the functional enrichment for glycolysis-related genes in ST expressing cells (Fig 1D and S3A Fig and S3 Table), we focused on characterizing the role of MCT1 (SLC16A1), a major monocarboxylate transporter that most closely followed the ST expression pattern. Genetic and pharmacological inhibition of MCT1 has been an effective strategy for targeting highly glycolytic tumors, inhibiting tumor growth through a combination of effects including accumulation of intracellular lactate, altering the production of glycolytic intermediates, reducing glucose transport and ATP levels, and reducing glutathione levels [33, 37, 41–43]. Cancer cells with elevated MCT1 expression have also been found to be exquisitely sensitive to the glycolysis inhibitor 3-bromopyruvate [44].

Through extracellular flux experiments, we found a significant increase in the ECAR of ST expressing IMR90 cells (Fig 3C), accompanied by increased sensitivity to MCT1 inhibition (Fig 3E). We observed that MCPyV-transformed IMR90 cells exhibited significantly higher ECAR levels compared to untransformed IMR90 cells (Fig 7B). Furthermore, MCPyV-transformed IMR90 cells (Fig 7D) and MCPyV-positive MCC cell lines (Fig 4) were sensitive to MCT1 inhibition. The sensitivity of these MCC cell lines to MCT1 inhibition corresponded to their relative ECAR levels. These results indicate that ST, together with LT, can manipulate cellular energy states to meet the demands of tumorigenesis.

We have demonstrated that ST plays a significant role in altering the metabolic state of the host cell, but our results do not rule out the possibility that further modulation by LT is required for its effect on transformation. The presence of MCPyV LT in the transformed cell experiments using PHE and PHEL cells could have an effect on the ability of ST to induce glycolysis genes. Previous transcriptional profiling of LT did not indicate any significant perturbation of the metabolic pathways being investigated here [22]. As MCPyV LT can still bind pRB, there could be perturbations of metabolic genes that cooperate with ST [45], as seen in PHE cells where HK2 and LDHA levels were increased compared to PH and ST-only cells (Fig 6B).

The regulation of MCT1 expression has been proposed to involve numerous factors, including MYC [33] and NF-κB [37]. Intriguingly, one study found that a significant number of MCC tumors contained genomic amplification of MYCL [40], a close relative of MYC that is also amplified in small cell lung cancer [46]. MYCN, another MYC isoform, is amplified in pediatric neuroblastoma and has been shown to regulate metabolic pathways in a similar way as MYC [47]. Given these findings, we investigated whether MYC, MYCN and MYCL could cooperate with MCPyV to regulate glycolysis gene expression.

By generating a series of dox inducible constructs expressing the MYC, MYCN and MYCL isoforms in both MCC cells and IMR90 lines, we found that MYC and MYCN could robustly affect the expression of MCT1 and the critical glycolysis enzyme HK2 (Figs 5A and 6B). We found that unlike IMR90 cells, MCC cell lines lacked endogenous MYC expression but did express MYCL and MYCN (S4A Fig). MYCL overexpression led to increased levels of MCT1 in MCC cells, but decreased levels in IMR90 PHE cells. This suggests that the observed lack of MYC expression in these MCC cells may alter the transcriptional activity of MYCL.

MCPyV ER expression independently upregulated LDHA expression in IMR90 cells in a manner not dependent on MYC signaling, whereas MYC affected LDHA expression in MKL-1 cells, suggesting that metabolic regulation by MCPyV may involve cell type-specific factors. Interestingly, PHEL cells had significantly lower ECAR than PHE cells (Fig 7B), although PHEL cells are fully transformed and are sensitive to specific MCT1 inhibitors (Fig 7C and 7D). This may be due to the observation that expression of MYCL in PHE cells decreased MCT1 expression (Fig 6B and 6C). Taken together, these results suggest that MYC and MYCN behave quite differently from MYCL, with MYCL appearing to have a particular synergy with MCPyV ST that influences both gene expression and transformation.

Soft agar experiments, besides testing for anchorage-independent growth, also place cells in an environment that likely has a lower diffusion rate for extracellular metabolites compared to the typical environment encountered on a plastic dish containing liquid growth media. While the effects of SR13800 and SR13801 are modest in the standard cell culture plate setting used in Fig 7C, the significant decrease in transformation shown in Fig 7D by these inhibitors is likely due to the fact that as a colony of cells grows in size in soft agar, it is forced to become more glycolytic as hypoxia increases. This increased glycolytic load, compounded with the inhibition of MCT1, may lead to toxic intracellular acidification that results in the significant colony formation defect.

Previous work from our lab and others has suggested that MCPyV ST has specific effects on NF-κB signaling [22, 48]. We found that MCT1 expression could be efficiently suppressed through RNAi knockdown of the canonical NF-κB subunit RelA in IMR90 PH and PHE cells (Fig 6C). Overexpression of MYC could still induce MCT1 in PHE cells following RelA depletion, although to a lower degree, suggesting that MYC is the primary regulator of MCT1 expression in these cells with NF-κB potentially having a supplemental role. These results agree with our promoter analysis (Fig 6A) and earlier studies indicating that MCT1 is regulated by multiple transcription factors [33, 37, 38]. Other transcription factors for genes encoding glycolytic enzyme, PPP enzymes and glucose transporters, such as the carbohydrate responsive element binding proteins (CHREBPs) [49], would be interesting candidates to pursue in future studies. We focused on the MYC family in this study as they are a major driver of glycolysis gene expression, have been implicated in the regulation of MCT1 and in particular, MYCL has been found amplified in MCC.

MCT1 primarily mediates intracellular or extracellular acidification depending on the cell and tumor type [27, 50]. Our data indicates that MCT1 inhibition in MCPyV-expressing IMR90 fibroblasts dramatically alters extracellular acidification. The effect of CHC compared to the more specific MCT1/2 inhibitors suggests that broad MCT inhibition may be more potent across MCC cell lines. This is supported by observations that MCT4 can also export lactate in a redundant fashion [51]. WaGa cells may have higher levels of MCT4 that could contribute to monocarboxylate transport and thereby limit viability defects from specific MCT1 inhibition, or could uniquely utilize lactate for energy production.

Our results here represent the first temporal transcriptome analysis of a DNA tumor virus protein, leading to the identification of key metabolic perturbations that contribute to the proliferative effects of MCPyV ST. We have also shown the differential effects of MYC, MYCN, MYCL and NF-κB on aerobic glycolysis and MCT1 regulation. Recent genetic analysis of patients with severe ketoacidosis identified several inactivating mutations in MCT1, correlating with disease severity, MCT1 protein levels and transport capacity, highlighting yet another critical role of this transporter in human health [52]. The key role of MCT1 in MCC viability should be considered in future treatment regimens, perhaps in combination with metformin or other metabolic agents that have previously shown promise when combined with MCT1 inhibition [51].

## Materials and Methods

### Cell culture

293T and IMR90 cells were obtained from ATCC. The MKL-1 and MKL-2 cell lines were kind gifts from Masahiro Shuda and Yuan Chang (University of Pittsburgh, PA), BroLi cells from Roland Houben (University of Wuerzburg, Germany) and WaGa cells were from Jürgen Becker (Medical University Graz, Austria). 293T cells were cultured in Dulbecco’s modified Eagle medium (DMEM) (Cellgro) supplemented with 1% Pen Strep (GIBCO), 1% Glutamax (GIBCO), and 10% fetal bovine serum (FBS) (Sigma). IMR90 cells were cultured with a similar media composition as the 293T cells with the exception of 15% FBS and addition of 1% non-essential amino acids (GIBCO). MCC cell lines were cultured in RPMI 1640 media (GIBCO) supplemented with 1% Pen Strep, 1% Glutamax, and 10% FBS.

Packaging and envelope plasmids were co-transfected with lentiviral or retroviral expression vectors into 293T cells using Lipofectamine 2000 (Life Technologies). Two days after transfection, 293T cell supernatant was clarified with a 0.45 μm filter and supplemented with 4 μg/mL polybrene (Santa Cruz) before transducing recipient cells. Stable cell lines were generated after selection with 2 μg/mL puromycin (Sigma), 5 μg/mL blasticidin (Invivogen), 500 μg/mL neomycin (Sigma) and 50 μg/mL hygromycin (Santa Cruz) as required by each vector. For inducible cell line experiments, doxycycline (Clontech) was used at 1 μg/mL. For MCT1 transport inhibitor experiments, dimethyl sulfoxide (DMSO) (Sigma), α-cyano-4-hydroxycinnamate (CHC) (Sigma) (5 mM), SR13800 (Calbiochem) (100nM), and SR13801 (Tocris) (100 nM) were used at the indicated concentrations.

### DNA

MCPyV ST, MYC-T58A, MYCN, MYCL and GFP Gateway-compatible cDNA entry clones were transferred from pDONR221 donor vectors to the pLIX_402 doxycycline inducible lentiviral Gateway destination vector (a gift from David Root; Addgene plasmid # 41394) via Gateway cloning (Life Technologies). pBabe-neo-p53DD was a gift from William C. Hahn (Dana-Farber Cancer Institute). pBabe-hygro-hTERT was a gift from Bob Weinberg (Addgene plasmid # 1773) [53]. Tumor-derived (MCCL21) MCPyV ER cDNA was generated as previously described [36] and cloned into the pLenti CMV Blast DEST (706–1) vector (a gift from Eric Campeau; Addgene plasmid # 17451) [54]. Lentiviral packaging plasmid psPAX2 and envelope plasmid pMD2.G were gifts from Didier Trono (Addgene #12260, #12259). Retroviral packaging plasmid pUMVC3 was a gift from Bob Weinberg (Addgene # 8449) [55] and envelope plasmid pHCMV-AmphoEnv from Miguel Sena-Esteves (Addgene # 15799) [56].

### RNAseq time course

Dox inducible IMR90 lines expressing ST or GFP were seeded in 6 cm dishes 24 hours before initiation of time course with dox-containing DMEM. Cells were harvested every 8 hours for 96 hours and total RNA was purified using RNeasy Plus Mini Kit (Qiagen) and mRNA was isolated with NEBNext Poly(A) mRNA Magnetic Isolation Module (New England BioLabs). IMR90 cells were refed after 48 hours with dox-containing media. Sequencing libraries were prepared with NEBNext mRNA library Prep Master Mix Set for Illumina (New England BioLabs), passed Qubit, Bioanalyzer and qPCR QC analyses and sequenced on HiSeq 2000 system (Illumina). The complete set of RNAseq data can be accessed from the Gene Expression Omnibus (GEO) repository GSE79968.

### Immunoblotting

The following antibodies were used: MCPyV Ab5 [36, 57]; GFP (D5.1, Cell Signaling); vinculin (H-10, Santa Cruz); actin (D6A8, Cell Signaling); HK2 (C64G5, Cell Signaling); LDHA (EP1565Y, Abcam); MCT1 (A1512, NeoBiolab); LAT1 (5347, Cell Signaling); RelA (D14E12, Cell Signaling); IκBα (L35A5, Cell Signaling); MYC (9E10, Santa Cruz); MYCN (9405, Cell Signaling); MYCL (AF4050, R&D Systems).

Whole cell lysates were prepared using RIPA buffer (Boston BioProducts) supplemented with protease inhibitor cocktail set I (Calbiochem) and phosphatase inhibitor cocktail set I (Calbiochem). Clarified protein extracts were boiled in SDS sample buffer (Boston BioProducts), resolved by SDS-PAGE (Criterion TGX precast gels; Bio-Rad), transferred to nitrocellulose membranes (Bio-Rad), blocked and incubated with the appropriate primary antibody in TBS-T overnight at 4°C. Detection of proteins was performed with horseradish peroxidase-conjugated secondary antibodies (Rockland), developed using Clarity Western ECL substrate (Bio-Rad), and imaged with a G:BOX Chemi detection system (Syngene).

### Real-time qPCR

Total RNA was purified using RNeasy Plus Mini Kit (Qiagen). cDNA was synthesized from the RNA using a High-Capacity cDNA Reverse Transcription kit (Thermo Fisher). qPCR was performed using Brilliant III SYBR Master Mix (Agilent Genomics) following the manufacturer’s instructions. RPLP0 was used as an internal loading control to normalize RNA levels.

### RNAi

IMR90 PH and PHE cells were seeded in 6 cm dishes (5 x 10^5^ cells/dish) and were transfected with 100 nM siRNA ON-TARGETplus SMARTpool siRNA against human RelA (L-003533-00-0005) or ON-TARGETplus Non-Targeting siRNA#1 (D0018100105) from GE Healthcare Dharmacon using Lipofectamine RNAiMAX (Life Technologies). After 24 hours, cells were refed with media with or without dox. Cells were then harvested for subsequent immunoblotting after 48 hours.

### Bioinformatic analysis

Reads were mapped to a transcriptome index generated from the hg19 human reference genome and the Merkel cell polyomavirus sequence, using Tophat 2.0.4 and Bowtie1 with default parameters. Novel junctions were not allowed. MCPyV-aligned reads were transformed into gene-level counts using HTSeq. Human-aligned reads were quantified at the gene level using Cufflinks 1.3.0 along with its ancillary algorithms that correct for biases and multi-mapped reads. Log-transformed FPKM (fragments per kilobase of transcript per million mapped reads) values were input into further statistical analysis of human transcript levels. All genes that had a zero FPKM value in any sample were deemed to have low expression and removed from the analysis.

The R package *limma* was used to rank genes by their differential expression in the ST cell line between every time point and the zero time point, relative to the same comparison in the GFP cell line [23]. We selected all genes with *P* < 0.01 and with total absolute fold change across all time points (relative to GFP) above a cutoff of 4. This resulted in a list of 2854 genes that were differentially perturbed over time by ST. We used the R package *mclust* to cluster the genes, based on their expression across all time points in the ST inducible cell line. Expression values were mean-centered and scaled by the standard deviation across the ST samples. The 50 clusters were tested for GO term enrichment using the R package *GOstats* with p-values adjusted for multiple testing by the Benjamini-Hochberg method. Similarly, the clusters were also tested for enrichment in pathways representing the hallmarks of cancer downloaded from the Molecular Signatures Database (MSigDB) [58]. The significance of each overlap was evaluated using a hypergeometric test and adjusted for multiple testing using Benjamini-Hochberg.

Position weight matrices (PWMs) for NFKB1 and MYC::MAX were extracted from the R package *MotifDB*. The SLC16A1 promoter from -1000 to +100 relative to TSS was mapped to the binding motifs using a cutoff of 0.8 for the estimated probability of a match between the promoter sequence and the PWM [59]. DNAse I hypersensitivity data for IMR90s was downloaded from the Gene Expression Omnibus (GEO), accession numbers GSM468801, GSM530665, GSM530666, and GSM468792.

### Metabolic analysis

Extracellular acidification rate (ECAR) was measured using the Seahorse Bioscience XFe24 Extracellular Flux Analyzer according to the manufacturer’s protocol. For IMR90 extracellular flux analysis, cells were seeded into assay culture plates (2 x 10^4^ cells/well) 24 hours prior to the assay. For MCC cell line analysis, assay culture plates were coated with Cell-Tak (Corning) following protocol from Seahorse Bioscience. Cells (1 x 10^5^ cells/well) were adhered to coated assay plate wells via centrifugation.

Cells were rinsed and cultured in XF Base Medium (Seahorse Bioscience) supplemented with 10 mM glucose (GIBCO), 1 mM sodium pyruvate (Sigma), 1% Glutamax, and pH was adjusted to 7.4 prior to performing the assay. Where described, DMSO and CHC were added to complete XF media before the start of the assay. Real-time OCR and ECAR data are representative of two biological replicates, with values representing the means and error bars representing standard deviation of five technical replicates at each time point.

For media glucose and lactate measurements, IMR90 cells inducibly expressing either ST or GFP were seeded in duplicate (1 x 10^5^ cells/well) in a 24-well plate using standard IMR90 culture media supplemented with 5 mM lactate (Sigma). Dox was added and media was collected daily for 5 days. Day 0 media corresponds to a sample of fresh growth medium. Glucose and lactate was measured using a YSI Biochemistry Analyzer.

### Anchorage-independent growth and proliferation assays

IMR90 anchorage-independent growth was performed as described [36] using 6-well dishes with SeaPlaque Agarose (Lonza) at concentrations of 0.3% top and 0.6% bottom layers. Agarose was diluted with 2X MEM (Gibco) supplemented with 2X Glutamax, 2X Pen Strep, and 30% FBS. IMR90 cells (10^4^) were seeded in triplicate in the top agarose layer. Wells were fed with top agarose twice per week. After 4 weeks, cells were stained with 0.005% crystal violet (Sigma) in PBS and colonies were counted. MCT1 inhibitors were included into soft agar layers at the concentrations described above.

IMR90 proliferation assays were performed as previously described [60]. Briefly, IMR90 cell lines were seeded in triplicate in 24-well plates (day 0; 5 x 10^3^ cells per well). Cell density was measured by crystal violet staining at intervals after plating as previously described [22]. MCC cell line proliferation assays were performed in triplicate in 48-well plates using XTT assay (Roche) following the manufacturer’s protocol.

## Supporting Information

S1 Fig Fold change of IMR90 RNAseq replicas and corresponding clusters. (TIFF)

S2 Fig Fold change in expression of SLC genes. (TIFF)

S3 Fig Fold change in expression of SLC genes ChREBP (A) and glutaminolysis (B) related genes after GFP and ST expression. (TIFF)

S4 Fig MYC isoform expression in IMR90 and MCC cell lines. **A)** IMR90 and MCC lines MKL-1, MKL-2, WaGa and BroLi were immunoblotted for the indicated MYC isoforms, ST and vinculin. **B-C)** IMR90 +/- ER with inducible expression of MYC, MYCL and MYCN and MKL-1 cells were assessed for MYCL expression by RT-qPCR.
(TIFF)

S1 Table Cluster membership for ST differentially expressed genes. (XLSX)

S2 Table GO term annotations for ST clusters. (XLSX)

S3 Table MSigDB annotations for ST clusters. (XLSX)
