# Supplementary figures and images for: Merkel Cell Polyomavirus Small T Antigen Promotes Pro-Glycolytic Metabolic Perturbations Required for Transformation
Source: PLoS Pathog. 2016 Nov 23;12(11):e1006020. doi: 10.1371/journal.ppat.1006020 (PMC5120958; doi:10.1371/journal.ppat.1006020)

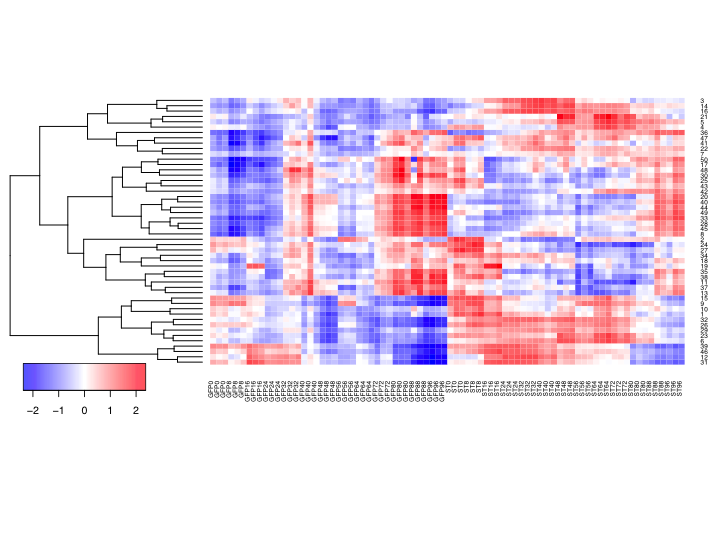

Supplement: S1 Fig — (TIFF) [file ppat.1006020.s001.tiff]

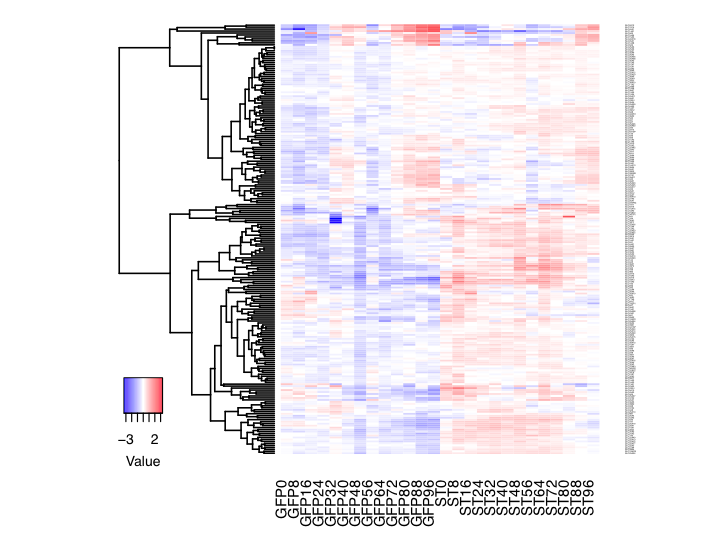

Supplement: S2 Fig — (TIFF) [file ppat.1006020.s002.tiff]

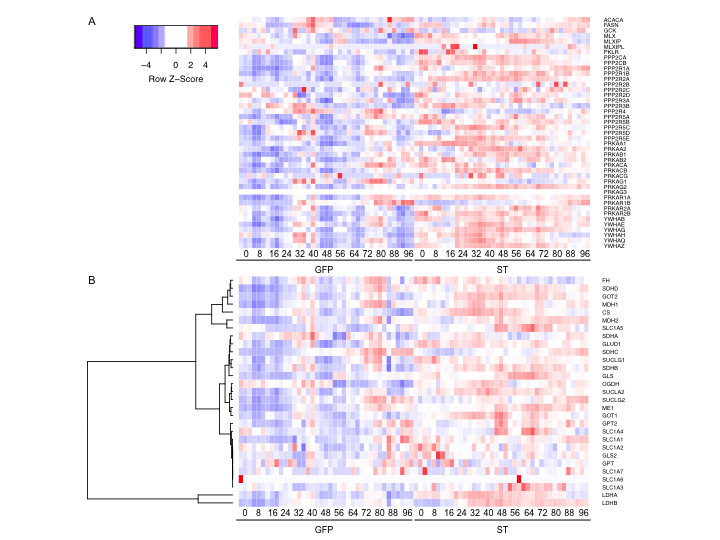

Supplement: S3 Fig — (TIFF) [file ppat.1006020.s003.tiff]

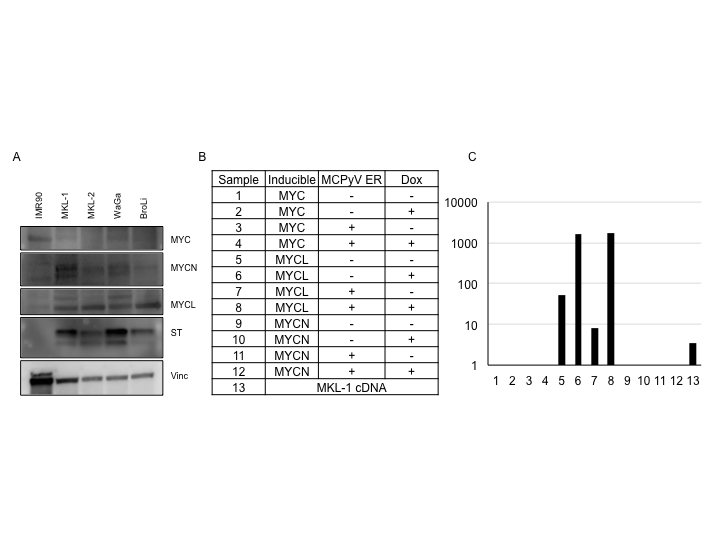

Supplement: S4 Fig — A) IMR90 and MCC lines MKL-1, MKL-2, WaGa and BroLi were immunoblotted for the indicated MYC isoforms, ST and vinculin. B-C) IMR90 +/- ER with inducible expression of MYC, MYCL and MYCN and MKL-1 cells were assessed for MYCL expression by RT-qPCR. (TIFF) [file ppat.1006020.s004.tiff]
